# Nucleocytoplasmic Shuttling of FTO Does Not Affect Starvation-Induced Autophagy

**DOI:** 10.1371/journal.pone.0168182

**Published:** 2017-03-13

**Authors:** Aleksander Aas, Pauline Isakson, Christian Bindesbøll, Endalkachew A. Alemu, Arne Klungland, Anne Simonsen

**Affiliations:** 1 Department of Molecular Medicine, Institute of Basic Medical Sciences, University of Oslo, Oslo, Norway; 2 Department of Molecular Microbiology A3.3021, Oslo University Hospital, Rikshospitalet, Sognsvannsveien 20, Oslo, Norway; Univerzitet u Beogradu, SERBIA

## Abstract

Polymorphic variants of the FTO (fat mass and obesity) gene associate with body mass index in humans, but the underlying molecular mechanisms have not been firmly determined. FTO is linked to energy homeostasis via amino acid sensing and is thought to activate the mammalian target of rapamycin complex 1, a negative regulator of autophagy. FTO localises both to the nucleus and the cytoplasm, and in this study we identify a functional nuclear localisation signal (NLS) in the N-terminus of FTO, as well as nuclear localization information in its very C-terminus. Inhibition of FTO nuclear transport has no effect on autophagy and in contrast to a previously proposed role of FTO in autophagy, we find no difference in starvation-induced autophagy in control cells compared to a panel of cell types depleted of FTO. Future studies that further characterise the cellular functions of FTO will be important to understand why variants in FTO are associated with body weight.

## Introduction

Obesity is becoming an increasing threat to the public health, growing into epidemic proportions [1]. While the heritability of fat mass is estimated to be between 40% and 70% since the 90`s, the candidate genes have been challenging to identify [2]. Genome-wide association studies (GWAS) have robustly linked single nucleotide polymorphisms (SNPs) within introns of Fat mass-and obesity-associated gene (*FTO)* with obesity and type 2 diabetes [3–5]. Although mouse models with different *Fto* expression levels confirm the effect of FTO on body composition [6–8], the underlying molecular mechanism remains elusive. Adding to the controversy around FTO, a recent report [9] clearly showed that the obesity associated SNPs in *FTO* function as a long-range promoter for the downstream *IRX3* (Iroquois Homeobox 3) gene, but not for *FTO*. Despite this, several reports suggest that FTO has a direct role in regulation of food intake and preference [10], as well as fat development, maintenance and metabolism [11–14]. Additionally, FTO has been linked to the activity of mammalian target of rapamycin complex 1 (mTORC1) and lack of FTO has been suggested to induce degradation or recycling of cellular components through autophagy [15].

Recombinant FTO can catalyse the demethylation of 3-methylthymine (3meT) to thymine in single-stranded DNA and 3-methyluracil (3meU) to uracil in RNA in a Fe(II) and 2-oxoglrutarate-dependent manner *in vitro* [16]. *N*^*6*^methyladenosine (6meA) in RNA is also a substrate for FTO with a 50-fold higher affinity than other substrates [17], indicating that FTO has a role in modifying RNA and DNA. FTO plays an important role in adipocyte differentiation [13], and the 6meA demethylase activity of FTO is required for preadipocyte differentiation [14].

While the crystal structure of FTO reveals an N-terminal catalytic domain and a C-terminal domain without any known functions [18], the signals involved in FTO localisation and possible nucleocytoplasmic shuttling has not been established. Linking the function of FTO to its subcellular localisation will be an important step forward. In this study we have identified an N-terminal NLS and a C-terminal nuclear localisation region in FTO. We show that the nucleocytoplasmic localisation of FTO has no effect on starvation-induced autophagy. In contrast to a previously proposed role of FTO in autophagy, we find no effect on autophagy in several assays using control cells compared to siRNA-mediated knockdown of FTO and *Fto*^-/-^ mouse embryonic fibroblasts (MEFs).

## Material and Methods

### Reagents

Rabbit monoclonal anti-FTO antibody (Epitomics, cat.no 5325–1); mouse monoclonal FTO-antibody (Cayman Chemical, cat.no 10816); mouse polyclonal FTO-antibody (Santa Cruz Biotech, cat.no sc-98769); mouse monoclonal FTO-antibody (Santa Cruz Biotech, cat.no sc-271713); rabbit monoclonal FTO-antibody (AbCam, cat.no ab126605); Mouse anti-β-actin and mouse anti-GFP (Sigma-Aldrich), guinea pig anti-p62 (Progen, cat.no GP62-C), mouse anti-p62 (BD, cat.no 610833), rabbit anti-LC3 (MBL cat.no PM036); mouse anti-HSP90 and mouse anti-GAPDH (AbCam, cat.no ab1429 and cat.no ab9484); Rabbit anti-Giantin (Covance, cat.no PRB-114C); rabbit anti-Histone H3 (AbCam, cat.no ab1791). HRP- or Cy2/3/4-conjugated secondary antibodies (Jackson ImmunoResearch Laboratories) and Alexa-conjugated secondary anitbodies (Invitrogen) were used. Bafilomycin A1 (BafA1, AH Diagnostics), an inhibitor of the lysosomal proton-ATPase, was used at 100 nM for 4 hours to block autophagy.

### Cell culture and transient transfections

HeLa, U2OS, HEKS293 and MEFs cells were maintained in Dulbecco`s Modified Minimal Essential Medium (DMEM) supplemented with 10% Fetal Bovine Serum (FBS), Dialysed Fetal Bovine Serum (Sigma Aldrich, F0392), 5 U/ml penicillin and 50 μg/ml streptomycin (P/S), hence called complete medium (CM). For starvation in nutrient-deplete medium, the cells were incubated in Earls Balanced Salt Solution (EBSS) either supplemented with dialysed FBS or not. Plasmids were transfected using Lipofectamine 2000 (Invitrogen) and 500 ng of plasmid was used for 24 hours. siRNAs (all used at 30 nM final concentration) were delivered to cells by Lipofectamine RNAi max (Invitrogen) for reverse transfection for 72 hours. All Cell lines used were purchased from American Type Culture Collection (ATCC).

### Fractionation

The Subcellular Protein Fractionation Kit for Cultured Cells was used as described by the manufacturer (ThermoFisher Scientific, Cat no. 78840), to fractionate U2OS cells.

### Mouse tissues and MEFs

All mice (C57BL6) were sacrificed by CO_2_ asphyxiation. Organs were isolated from mice age 10–12 weeks and primary MEFs were isolated from embryos taken out at age E13.5 after *Fto*^+/-^ x *Fto*^+/-^ breeding. MEFs were cultured for several passages until they became immortalized spontaneously. The tissues were disrupted with TissueRuptor using TissueRuptor Disposable Probes (QIAGEN, cat no. 990890) following the protocol described in the kit. From this we isolated RNA and made protein lysates. All mouse experiments were approved by the Section for Comparative Medicine at Oslo University Hospital and by the Norwegian Animal Research Authority. The work was conducted in accordance with National laws and Federation for Laboratory Animal Science Associations (FELASA) regulations.

### Long-lived protein degradation

To measure long-lived protein degradation by autophagy, cells were labelled with 0.25 μCi/ml L-[^14^C] Valine (Perkin Elmer) for 24 h in CM, then washed in CM and chased for 16 h in CM supplemented with 10mM valine (Sigma-Aldrich) to allow short-lived protein degradation. After a wash in EBSS or CM, cells were starved (in EBSS containing 10mM cold valine) or not (in CM containing 10mM cold valine) in the presence or absence of 100 nM BafA1 for 4 h. Following the treatment, the cell media was transferred to Eppendorf tubes and 10% trichloroacetic acid (TCA) was added to precipitate proteins. 0.2 M KOH was added to cells remaining in the dish. All samples were incubated at 4°C overnight. 3 mL Ultima Gold LSC cocktail (PerkinElmer) was added to samples and protein degradation was determined by measuring the ratio of TCA-soluble radioactivity to total radioactivity by a liquid scintillation analyser (Tri-Carb 3100TR; PerkinElmer). Percent degradation was defined as the acid-soluble radioactivity into the medium divided by the total radioactivity.

### Western blotting

Cells was first washed two times in PBS, then lysed in RIPA buffer (50mM Tris-HCl pH 7.5, 150 mM NaCl, 1 mM EDTA, 1% NP40, 0.5% Triton-X-100), containing complete protease inhibitor cocktail (11873580001; Roche) and PhosSTOP phosphatase inhibitor cocktail (4906837001; Roche). Protein concentrations were measured by a BCA protein assay (Bio-Rad laboratories) to run equal amounts of cell lysates on pre-casted 4–20% gels with SDS-PAGE, before transfer to PDVF membrane (Millipore). The membrane was blocked using 5% milk for 1 hour before incubation in the specified primary antibodies overnight and HRP-conjugated secondary antibodies, which were detected by the Supersignal West Dura Extended Duration Substrate kit (Pierce), or far-red fluorophore-conjugated secondary antibodies (LI-COR), detected with the Odyssey imaging system (LI-COR). Image J software was used for quantification.

### Fluorescence microscopy

Cells were grown on coverslips, treated as described. They were washed in cold PBS twice and fixed in 4% PFA for 15 min on ice. The cells were then permeabilised in a solution containing 5% FBS and 0.05% saponin (Sigma-Aldrich) in PBS before incubation with primary antibody for 1 hour. This followed by washing in cold PBS and incubation with secondary antibody for 45 min. Prolong Gold containing DAPI was used as mounting media (Life Technologies). Confocal microscopy was performed using Zeiss LSM 710 ELYRA. For quantitative analysis, cells were imaged on a Zeiss Cell Observer microscope. The AXIOVISION soſtware (Zeiss) was used for automated capture of 25 images (5 × 5) per sample. The physiology module of the ASSAYBUILDER soſtware (Zeiss) was used to identify suitable cells using the nuclear staining. GFP positive cell were then identified in GFP channel and the DsRed channel was used to identify discrete puncta in the GFP positive cells. Using manual setting of threshold values for gating of appropriate puncta size, shape and intensity, the soſtware then determined the number of puncta per cell and for all samples.

### qPCR

Total RNA from mouse tissue samples and siRNA-transfected cells were isolated with the RNeasy plus kit (QIAGEN) according to manufacturer’s instructions. 500 ng cDNA was synthesised by reverse transcription (iScript; Bio-Rad technologies), qPCR was performed by using SYBR green (QIAGEN) and primers sets for the indicated targets relative to a housekeeping gene (Tbp) on a Bio-Rad CFX analyser. Primers: TBP (Qiagen QT00000721), Fto (Qiagen QT00105427), Tbp (Qiagen QT00198443), Lc3a (Qiagen QT00284039), Lc3b (Qiagen QT01750322), Atg5 (QT100114751), p62 (Qiagen QT00127855).

### Construction of plasmids and mutagenesis

Different constructs of FTO and FTO fragments were generated by PCR from FTO full length cDNA. Mutated constructs were made with QuickChange II Site-Directed Mutagenesis Kit (Agilent Technologies). All plasmids were sequenced to ensure correct reading frame and to verify the presence of the desired mutations. Primers used for deletion constructs: 5`FTO-32 ATAGTCGACACCCCCAAAGATGATGAATTC, 3`FTO-495 ATTGCGGCCGCCTAGAGTTCTGAAACGATGTCTGT, 3`FTO-485 ATTGCGGCCGCCTACGGCAGAGGCATCGAAGCATCA 3`FTO-475 ATTGCGGCCGCCCAGTATGGCCGACATTCTGG, 3`FTO-445 ATTGCGGCCGCCTGGCGTGCAGTGAGCGAGGC, 3`FTO-415 ATTGCGGCCGCAGCATTTGTCACACCCTCCAT, 3`FTO-385 ATTGCGGCCGCGCCTTGAAACCAAAACTGCCT, 3`FTO-355 ATTGCGGCCGCCAAAGAGACATCATCATTGTC. Primers used for mutagenesis: 5`FTO KKLR->AALR TTCAAGAAGCCTCAGTGCCGCAGCTTCGCG, 3`FTO KKLR->AALR CGCGAAGCTGCGGCACTGAGGCTTCTTGAA

### Statistics

Averages of each value is calculated and the variation between experiments is shown by standard error of the mean (SEM) or Standard deviation (SD). Significance was calculated using a two-tailored student *t*-test. None of the tested parameters was found to be significant with a p-value less than 0.05.

## Results

### FTO expression pattern and subcellular localisation

**Fig 1:**
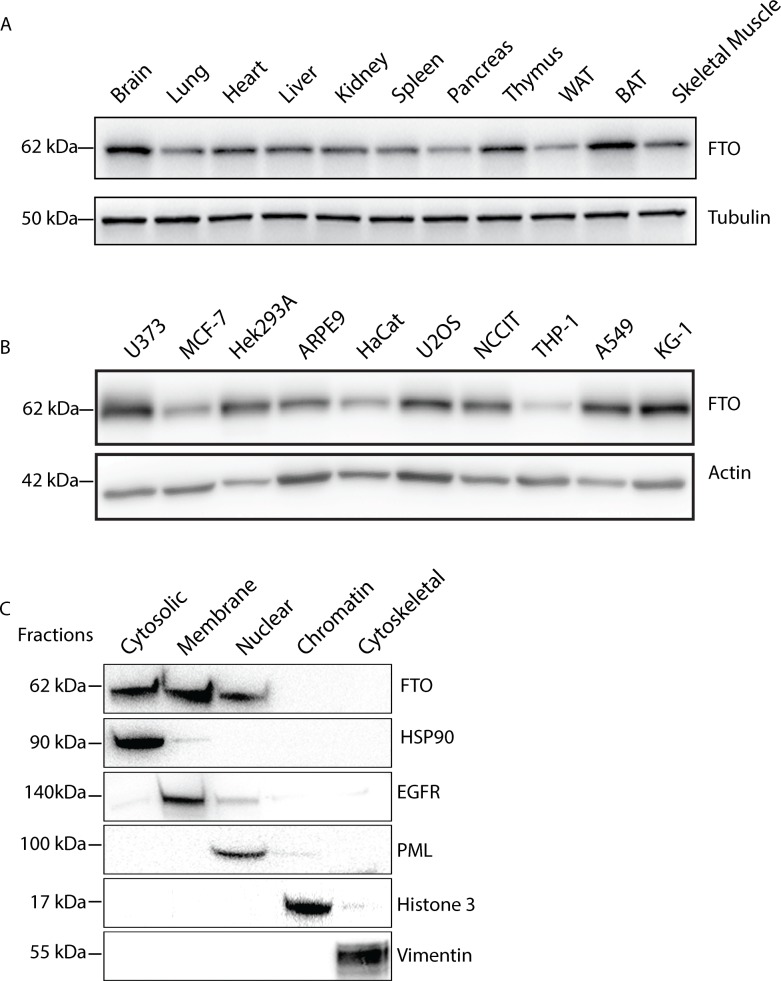
FTO is ubiquitously expressed. (A) Western blot analysis of the indicated mouse tissues probing for Fto using antibody from Epitomics (see Table 1). BAT; brown adipose tissue, WAT; white adipose tissue. (B) Protein extracts from the indicated human cell lines were analysed by immunoblotting using mouse anti-β-actin and rabbit anti-FTO (Epitomics) antibodies. (C) Various subcellular fractions of U2OS cells were immunoblotted using rabbit anti-FTO (Epitomics), anti-HSP90, anti-EGFR, anti-PML, anti-Histone-3 and anti-Vimentin. One representable experiment (out of two) is shown.

In order to study the cellular distribution and function of FTO we first determined the relative expression of FTO in different mouse tissues and human cell lines. The Fto protein was ubiquitously expressed in the panel of mouse tissues, with the highest expression in the brain, thymus and brown adipose tissue and the lowest expression in pancreas and white adipose tissue (Fig 1A). Accordingly, Fto mRNA expression was also found to be ubiquitous, with highest transcript levels in the skeletal muscle and pancreas, and similar transcription levels in the other tissues (S1 Fig). FTO was expressed in various human cancer cell lines, with the highest expression in U373, HEK293, A549 and KG-1, and lowest expression in THP-1, HaCat and MCF-7 when normalised to β-actin (Fig 1B).

The subcellular localisation of FTO is not fully understood. Some studies report FTO to be exclusively nuclear [17,19,20], whereas other report FTO to be exclusively cytoplasmic or both in the cytoplasm and nucleus [21]. We used a fractionation assay to determine the subcellular localisation of FTO in U2OS cells and found that FTO resides in the cytosol, as well as in the nuclear and membrane fractions (Fig 1C). Taken together, our data show that FTO is an ubiquitously expressed protein localised both in the nucleus and cytoplasm.

### FTO isoforms

When staining for endogenous FTO using different antibodies we discovered that some antibodies showed cytoplasmic staining of FTO, whereas others stained FTO in the nucleus (S2 Fig). All antibodies were specific as shown by reduced levels of FTO in cells treated with siRNA targeting FTO compared to scramble control cells (S2 Fig). Interestingly, the antibodies raised against the C-terminal region of FTO or the full length protein displayed mainly cytoplasmic FTO staining (S2A i, ii and iv Fig, Table 1), whereas the antibody recognizing an N-terminal epitope showed nuclear staining (S2A iii Fig, Table 1), suggesting that the antibodies may detect different isoforms that are localised to the nucleus and/or the cytoplasm. Using the FTO antibodies specified in Table 1 for immunoblotting of lysates from control or FTO depleted U2OS cells, we find however that all antibodies recognize the predicted full length FTO isoform at 62 kDa (except for the antibody from Cayman, which also detects a band around 80 kDa) (S3 Fig). By analysing the expression profiles of different FTO isoforms using the GTEx portal (http://www.gtexportal.org/home/gene/FTO)[22] we found that isoforms lacking the N-terminal region are the predominantly expressed isoforms in various human tissues. This suggests that there could be tissue specific FTO isoforms exerting different functions depending on their cellular localisation.

**Table 1:** List of the different FTO antibodies used.

Number	Name	Specie	Clonality	Cat #	Supplier	Binding	WB	IF
**i**	Fatso (H-300) Antibody	Mouse	Poly	sc-98768	Santa Cruz	FL	+	+
**ii**	FTO Antibody	Mouse	Mono	10816	Cayman	CT	+	+
**iii**	Anti-FTO antibody	Rabbit	Mono	Ab126605	AbCam	NT	+	+
**iv**	Fatso (C-3) Antibody	Mouse	Mono	sc-271713	Santa Cruz	CT	+	+
**v**	FTO	Rabbit	Mono	5325–1	Epitomics	CT	+	-

### FTO contains a functional nuclear localisation signal

**Fig 2:**
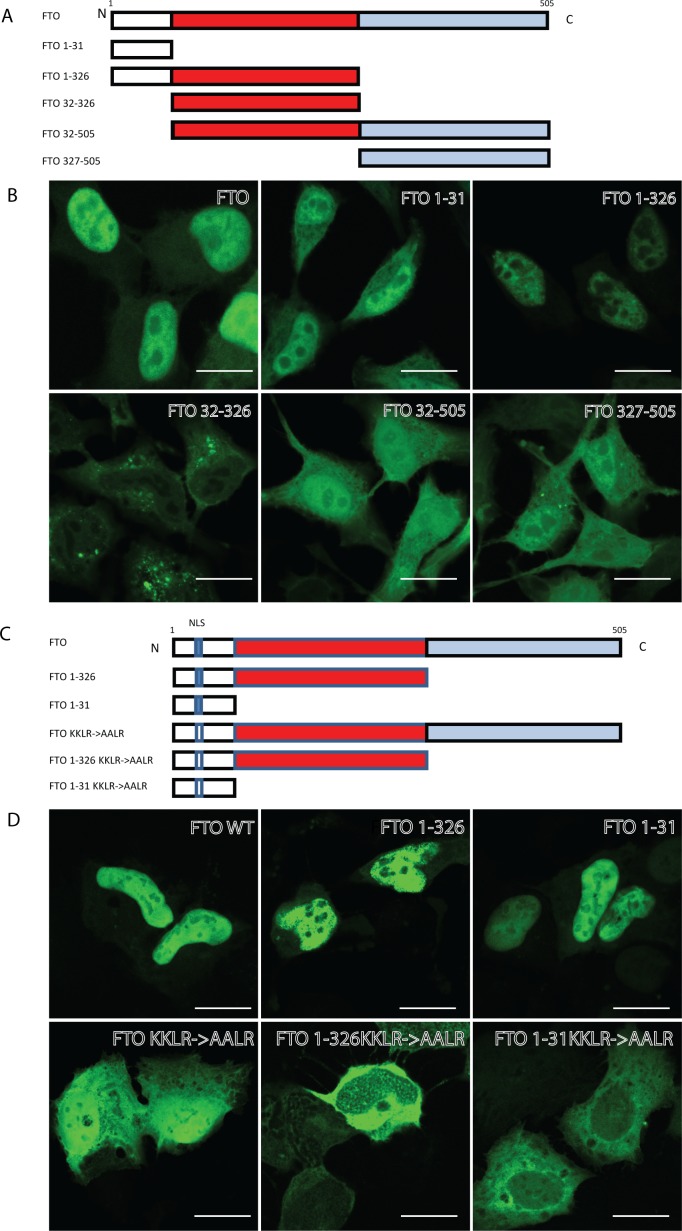
FTO contains a functional NLS. (A) Graphical description of the constructs used. Red denotes the N-terminal Fe(II) and 2-Oxoglutarate dependent Dioxygenase domain. Blue denotes the C-terminal domain without any known function. (B) Confocal microscopy images of HeLa cells transiently transfected with the denoted GFP-tagged constructs. (C) Graphical description of the constructs used. KKLR-> AALR denotes mutation of lysine residues to alanine residues in amino acid number 15 and 16 in the canonical sequence. (D) Confocal images of U2OS cells transiently transfected with the denoted constructs. Scale bar 20 μm.

FTO has been predicted to contain a nuclear localisation signal (NLS) in the N-terminus (amino acid (aa) position 2–18) [23], but this has not been shown experimentally and no functionality of this sequence has been reported. To determine if the proposed NLS is functional, we created full length and various truncated FTO plasmids. HeLa and U2OS cells were transfected with GFP-tagged full-length FTO (aa 1–505) or various FTO deletion mutants (Fig 2A and 2B) and their subcellular localisation determined by confocal imaging. While full-length FTO was mainly detected in the nucleus, deletion of the N-terminal 31 aa (FTO 32–326 and FTO 32–505) resulted in a cytoplasmic localisation of FTO. Indeed, expression of the first 31 aa alone (FTO 1–31), which are N-terminal to the catalytic domain and contains the predicted NLS, was sufficient for nuclear localisation of FTO (Fig 2B).

As the N-terminal 31 aa contains information for nuclear localisation of FTO we looked for a classical NLS in this stretch of amino acids and found the sequence KKLR in aa 15 to 18 (Fig 2C), which corresponds to nucleotide 264–276 of the canonical human FTO (UniProtKB Accession #Q9COB1).We preformed site directed mutagenesis to change the two first lysine residues to alanine residues (KKLR->AALR) in the constructs containing the putative NLS (FTO 1–505, FTO 1–326 and FTO 1–31).

Indeed, all the transfected KKLR->AALR mutant constructs lost the predominant nuclear localisation of FTO (Fig 2D), indicating that the motif KKLR is a functional NLS and important for the nuclear localisation of FTO.

**Fig 3:**
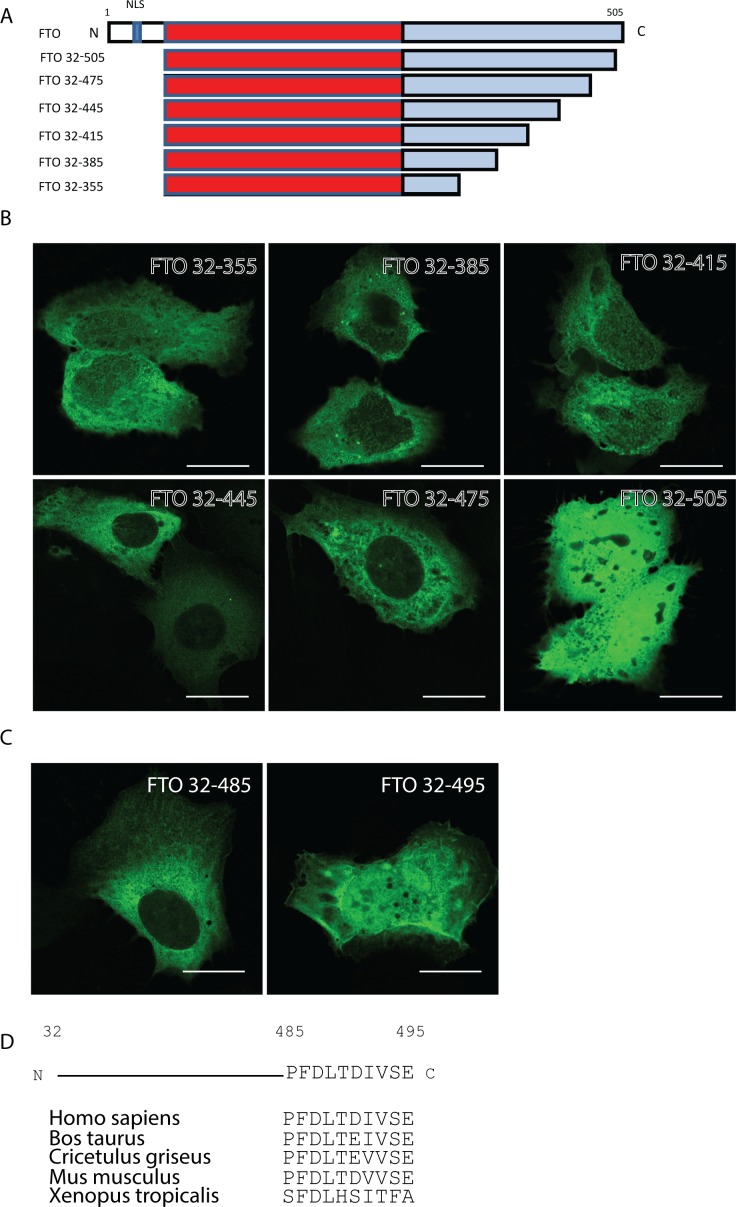
The extreme C-terminal part of FTO facilitates nuclear shuttling. (A) Graphical description of the constructs used. The numbers denotes the amino acids the constructs contain. (B) Confocal microscopy images of U2OS transiently transfected with the denoted GFP-tagged constructs. Scale bar 20 μm. (C) Confocal microscopy images of U2OS transiently transfected with the denoted GFP-tagged constructs. Scale bar 20 μm. (D) Diagram of the construct FTO 32–495 highlighting the 10 amino acid necessary for nuclear shuttling and an alignment of amino acid 486–495 in the denoted species.

Interestingly, ectopic expression of FTO with different constructs lacking the NLS, but containing the C-terminal region (aa 326–505), still showed a partial nuclear localisation of FTO (FTO 32–505, FTO 327–505, FTO KKLR→AALR). We found no typical NLS in this region using conventional bioinformatics tools, so we created deletion constructs lacking the N-terminal 31 aa and having progressive truncations from the extreme C-terminal part (Fig 3A). When these constructs were transfected in U2OS cells, we found that the C-terminal 30 aa of FTO contained additional sequence information responsible for nuclear localisation of FTO as FTO 32–475 localized exclusively to the cytoplasm (Fig 3B). By creating and transfecting two additional C-terminal FTO constructs (aa 32–485 and aa 32–495) we were able to narrow down the C-terminal nuclear localisation signal to aa 486–495 (Fig 3C). Sequence alignment of aa 486–495 revealed that this region is highly conserved from Xenopus Tropicalis to Homo sapiens (Fig 3D), suggesting an evolutionarily conserved function, such as nucleo-cytoplasmic shuttling.

Taken together, we report a functional NLS in the N-terminal region of FTO, which explains the strong nuclear staining of overexpressed full-length canonical FTO. We also uncover that the extreme C-terminal of FTO have the ability to shuttle FTO to the nucleus. Surprisingly, there seems to be a discrepancy in the localisation of endogenous and overexpressed FTO. This may be explained by the preferential expression of FTO isoforms lacking the N-terminus in human cells.

### FTO has no effect on autophagy

**Fig 4:**
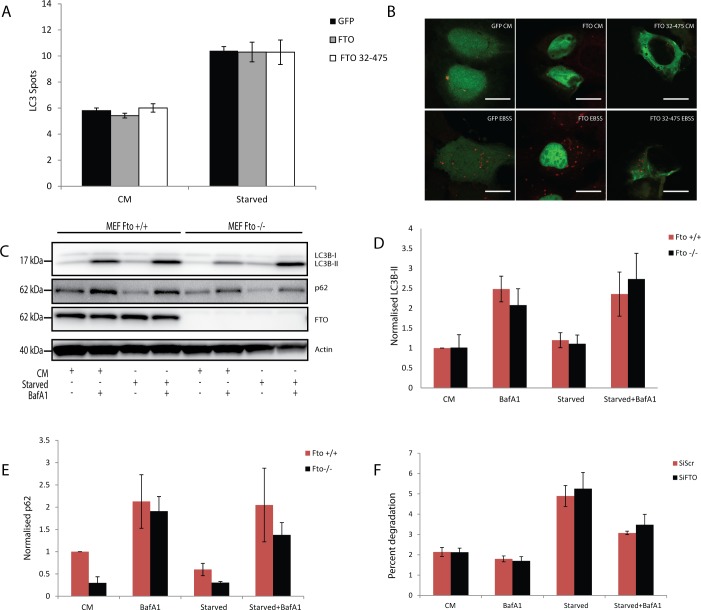
FTO has no significant effect on autophagy. (A) Quantification of LC3B puncta in cells transfected with GFP, full length GFP-FTO or GFP-FTO 32–475, treated either with complete media (CM) or EBSS starvation media (Starved). >1000 cells counted in each condition. Data shown is mean ± SEM. (B) Representative images of cells used for quantification of LC3B puncta, shown in red. Scale bar 20 μm. (C) Western blot analysis of protein lysates from *Fto*^+/+^ MEFs and *Fto*^-/-^ MEFs treated either with complete media (CM), complete media and BafA1 (BafA1), EBSS starvation media (Starved) or EBSS starvation media and BafA1 (Starved + BafA1). All treatments were for 4 hours. The blot is representative for five experiments. (D) Quantification of LC3B-II normalised to beta-actin and to CM *Fto*^+/+^ from five experiments as shown in A. The graphs show mean ± SEM (E) Quantification of p62 normalised to beta-actin and to CM *Fto*^+/+^ from three experiments as shown in A. The graphs show mean ± SEM. (F) Long-lived protein degradation in HeLa cells treated as in A. The graph shows results from five experiments, presented as mean ± SEM.

One of the proposed roles of FTO is that it is an amino acid sensor, which when depleted will give a signal of nutrition deprivation, leading to induction of autophagy [15]. We set out to test if the cellular localisation of FTO was important for its proposed role in autophagy. LC3B is an ubiquitin-like protein that upon induction of autophagy becomes cleaved and conjugated to phosphatidylethanolamine (PE) in the autophagic membrane and is therefore a widely used marker to study autophagy. U2OS cells were transfected with GFP, full length FTO or FTO 32–475 (which has no nuclear localisation), followed by induction of autophagy by incubation in starvation media for 4 hours and immunoblotting for LC3B. As expected, LC3B puncta accumulated upon starvation compared to cells incubated in control media (CM), but there was no significant difference between GFP transfected control U2OS cells and cells transfected with full length FTO (nuclear) or the cytoplasmic FTO 32–475 (Fig 4A and 4B). Thus, the cellular localisation of FTO seemed redundant for a putative role of FTO in starvation-induced autophagy.

Next we determined if cellular depletion of FTO was important for autophagy using Fto knockout mouse embryonic fibroblast (*Fto*^-/-^ MEFs) and several human cell lines transfected with FTO siRNA. The non-lipidated form of LC3B (LC3-I) can be separated from the lipidated form (LC3-II) by immunoblotting and the flux in the pathway can thus be quantified in the absence or presence of the lysosomal proton-pump inhibitor BafA1, which inhibits lysosomal degradation of LC3-II. The cells were subjected to starvation, with or without 100nM BafA1. MEFs from *Fto*^-/-^ mice and wild type (*Fto*^+/+^) littermates were used to investigate whether Fto is important for autophagic flux during normal or starved conditions. First we did a time course starvation where the cells where either incubated in CM for 6 hours or in EBSS containing dialysed serum for 0.5 hours, 2 hours, 4 hours or 6 hours with and without BafA1 (S4 Fig). Here we see that there is an adequate starvation induced degradation of LC3-II and that there is no real difference between *Fto*^+/+^ and *Fto*^-/-^ at any time point. Further we did not detect any significant differences in LC3-II levels between *Fto*^+/+^ and *Fto*^-/-^ MEFs in normal or starved conditions, with or without BafA1 treatment (Fig 4B and 4C) after 4 hours in EBSS not containing dialysed serum. Additionally, there was no difference in the level of the autophagy receptor p62/SQSTM1, which is itself a cargo for autophagy [24], but a non-significant trend of less p62 was observed in the *Fto*^-/-^ cells (Fig 4D). To further elucidate a possible effect of Fto on autophagy, the rate of degradation of long-lived proteins (known to be degraded by autophagy) was measured using the long-lived protein degradation (LLPD) assay. This assay is based on calculation of the difference between acid-soluble (degraded amino acids) and acid-precipitated (intact proteins) radiation in ^14^C-valine-labeled cells, providing an indication on the rate of autophagy. There was no significant difference in the percent degradation of long-lived proteins between *Fto*^+/+^ vs *Fto*^*-/-*^ MEFs (S5A Fig). Together, these results suggest that FTO has no role in starvation-induced autophagy in MEFs.

Next we questioned if FTO was important for autophagy in different human cell lines. The cells were depleted of FTO using a siRNA based approach, and then subjected to starvation or BafA1 treatment as explained above. FTO was depleted in HeLa, U2OS or HEK293 cells, followed by immunoblotting of cell lysates for LC3B and p62. Consistent with the results in MEFs, we did not observe any difference in the levels of these autophagy markers in cells depleted of FTO (S5C–S5E Fig). Further, there were no differences in the degradation of long-lived proteins between control cells and FTO depleted cells (Fig 4E and S5B Fig). By using confocal immunofluorescence microscopy we also did not detect any colocalisation of FTO with endogenous LC3B puncta in CM or starved HeLa cells, treated with BafA1 or not (S6 Fig). Collectively, these results suggest that FTO has no significant effect on starvation-induced autophagy.

## Discussion

Based on data from this study and previous studies we conclude that FTO is expressed in numerous tissues, including the brain and brown adipose tissue. This expression correlates well with the proposed function of FTO in the hypothalamic region [20,25] and as a regulator of adipogenesis [13,14]. We also show that FTO is expressed in a range of different human cancer cell lines, which may be related to a proposed role for FTO in regulation of genes related to cancer [26]. FTO is able to demethylate both RNA and DNA and it is therefore important to determine the cellular localisation of FTO. Our data show that endogenous FTO resides in the cytosolic, membrane and nuclear fraction of human U2OS cells. Earlier reports show that ectopically expressed FTO shuttles between the nucleus and cytoplasm [21] which is supported by our finding that endogenous FTO resides in all subcellular fractions.

In this study, we identify a functional NLS in the N-terminus of FTO (aa 15–18), which is sufficient to shuttle FTO to the nucleus. Interestingly, we also identified a C-terminal region of FTO (aa 485–495) that contains nuclear transport information. This region is highly conserved among species, but contains no typical NLS, no amino acids predicted to be modified by phosphorylation (phosphosite.org) and no important domain structure was found using Motif scanner. FTO constructs containing this signal alone (without the N-terminal NLS) localise both to the nucleus and the cytoplasm. Given that the C-terminal and polyclonal antibodies of FTO mainly show cytoplasmic staining, it is possible that the N-terminal NLS is not present and/or functional in all endogenous isoforms of FTO or that its function is context and tissue dependent. The GTEx portal reveals that the FTO isoforms that are predominantly expressed in human cells and tissues lack the N-terminal NLS, but contain the C-terminal nuclear transport signal, thus retaining the ability to shuttle to the nucleus. These isoform transcripts are suggested to be protein coding as revealed in ensemble.org. FTO has been suggested to have a key role in adipocyte differentiation and adipogenesis, but as FTO is ubiquitously expressed it is likely to also have other functions. Dissecting the roles of different FTO isoforms in different tissues and addressing how the nucleocytoplasmic shuttling of FTO relates to its function are important steps to further characterise the cellular functions of this protein.

FTO is a proposed amino acid sensor which can enhance mTORC1 activity [15,27]. mTORC1 is a negative regulator of autophagy [28] and thus, activation of the FTO-mTORC1 axis is suggested to inhibit autophagy [15,29]. In an attempt to address the significance of the nucleocytoplasmic shuttling of FTO, we took advantage of the proposed role of FTO in autophagy. Ectopic expression of nuclear or cytoplasmic FTO did not influence autophagic flux as measured by LC3 puncta in U2OS cells. Interestingly, we also found no role of endogenous FTO in autophagy using a panel of different cell lines and using several autophagy assays, such as p62 or LC3B protein levels, LC3B-lipidation or degradation of long-lived proteins. The subtle reduction of p62 protein levels in *Fto*^-/-^ vs *Fto*^+/+^ MEFs could be due to FTO affecting the turnover of p62 mRNA by stabilising it, but we did not detect any significant difference in mRNA levels of p62, Lc3A, Lc3B and Atg5 between *Fto*^-/-^ vs *Fto*^+/+^ MEFs (S5F Fig). Our data on autophagy are in contrast to what was reported by Gulati and co-workers, presenting immunoblots showing elevated LC3-II levels in *Fto*^-/-^ compared to *Fto*^+/+^ MEFs [15]. The observed difference could be explained by distinct intervariability differences between the MEFs used in these studies, the time of starvation used, or the high dose of BafA1 used by Gulati *et al*. In their study they used 400 μM BafA1, which is 4000 higher than the standard dose of 100 nM used in our study. We found that the 400 μM dose was toxic in our experimental conditions. Moreover, quantitation of LC3B levels is completely missing in the Gulati study.

In conclusion, we here identify an N-terminal NLS and a C-terminal nuclear transport signal in FTO, which both allows FTO to shuttle between the nuclear and cytoplasmic compartments. The localisation and expression of FTO is not changed by short-term amino acid starvation or by manipulation of autophagy, and expression and localisation of FTO is not affecting starvation induced autophagy. We show that FTO is not important for autophagy in response to starvation in mouse or human cell lines. Future studies aimed at characterising the function of FTO in different tissues and the roles of different FTO isoforms will hopefully lead to a better understanding of this protein and elucidate the mechanisms underlying the correlation between SNPs in FTO and obesity and type 2 diabetes.

## Supporting Information

S1 Fig Expression of Fto in different mouse tissues. The graph is showing the relative expression of Fto in the denoted tissue measured by real-time PCR and normalised to TATA box binding protein (Tbp). Data presented as mean ± SD.
(TIF)

S2 Fig Antibodies specific for FTO are showing different staining. (A) U2OS cells transfected with control or FTO siRNA were fixed and stained with different antibodies against FTO. The letters corresponds to the antibody used as shown in Table 1. (B) Western blot analysis of HeLa cell protein lysate showing knockdown of FTO. Scale bar 40 μm.
(TIF)

S3 Fig Antibodies specific for FTO in western blots. U2OS cells were treated with siControl or siFTO and stained with the antibodies specified in Table 1.
(TIF)

S4 Fig Time course starvation assay in MEFs. (A) Western blot analysis of MEFs treated with CM or EBSS supplemented with dialysed serum for 0.5h, 2h, 4h, or 6h with or with BafA1. BafA1 was added for 6 hours to the CM treated cells. (B) Quantification of LC3-II levels in MEFs treated with CM or EBSS supplemented with dialysed serum for 0,5h, 2h, 4h, or 6h with or with BafA1. Data are from 3 experiments and presented as mean ± SEM.
(TIF)

S5 Fig Depletion of FTO has no effect on autophagy. (A) Degradation of long-lived proteins in Fto^+/+^ or Fto^-/-^ MEFs treated either with complete media (CM), complete media (CM) and Bafilomycin A1 (BafA1), EBSS starvation media (Starved) or EBSS starvation media and Bafilomycin A1 (Starved + BafA1) for 4 hours. The data are from 2 experiments and presented as mean ± SD. (B) Degradation of long-lived proteins in U2OS cells treated as in A. The data are from 2 experiments and presented as mean ± SD. (C) Western blot analysis of protein lysates from control and FTO depleted HeLa cells treated either with complete media in the absence or presence of BafA1 for 4 hours. (D) Western blot analysis of protein lysates from control and FTO depleted U2OS cells treated as in C. (E) Western blot analysis of protein lysates from control and FTO depleted HEK293 cells treated as in C. (F) The graph is showing the relative expression of the denoted targets measured by real-time PCR and normalised to TATA box binding protein (Tbp) in MEFs. Data presented as mean ± SD.
(TIF)

S6 Fig FTO is not localised to LC3B-positive membranes. HeLa cells were starved (EBSS) or not (fed) in the absence or presence of BafA1 and then stained with antibodies against FTO (Cayman, Table 1) and LC3B. Scale bar 20 μm.
(TIF)
