# Supplementary figures and images for: Nucleocytoplasmic Shuttling of FTO Does Not Affect Starvation-Induced Autophagy
Source: PLoS One. 2017 Mar 13;12(3):e0168182. doi: 10.1371/journal.pone.0168182 (PMC5347998; doi:10.1371/journal.pone.0168182)

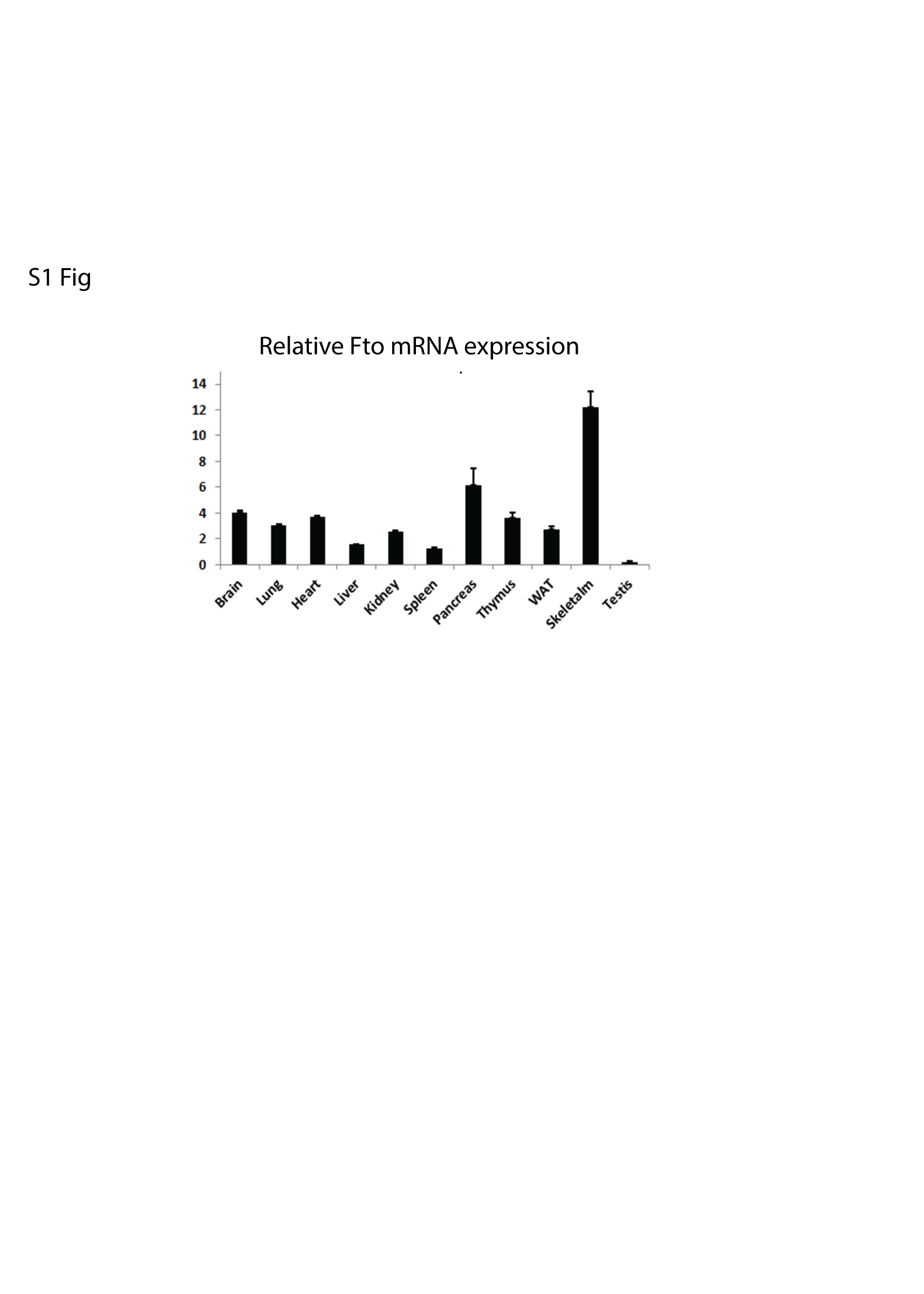

Supplement: S1 Fig — The graph is showing the relative expression of Fto in the denoted tissue measured by real-time PCR and normalised to TATA box binding protein (Tbp). Data presented as mean ± SD. (TIF) [file pone.0168182.s001.tif]

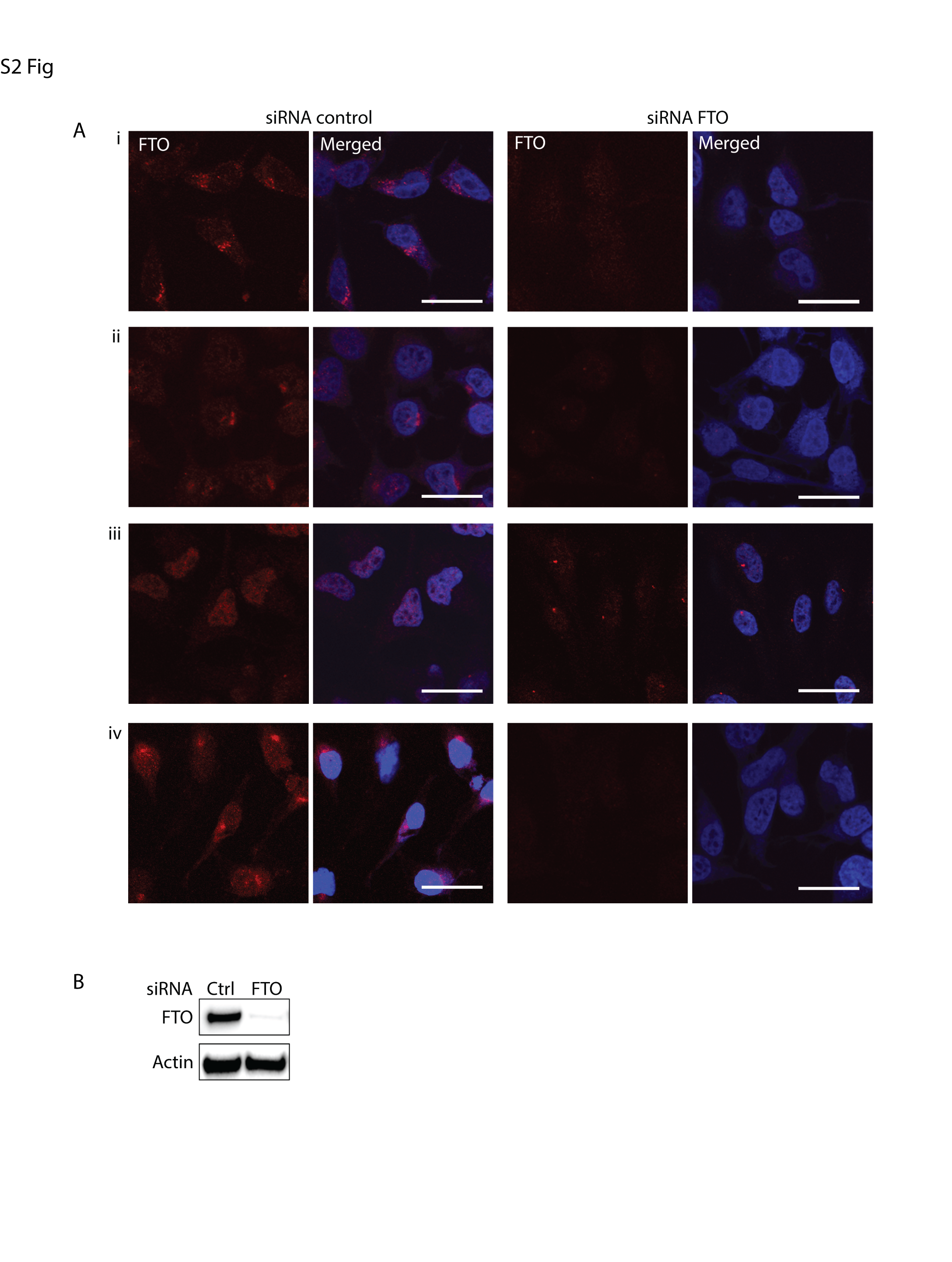

Supplement: S2 Fig — (A) U2OS cells transfected with control or FTO siRNA were fixed and stained with different antibodies against FTO. The letters corresponds to the antibody used as shown in Table 1. (B) Western blot analysis of HeLa cell protein lysate showing knockdown of FTO. Scale bar 40 μm. (TIF) [file pone.0168182.s002.tif]

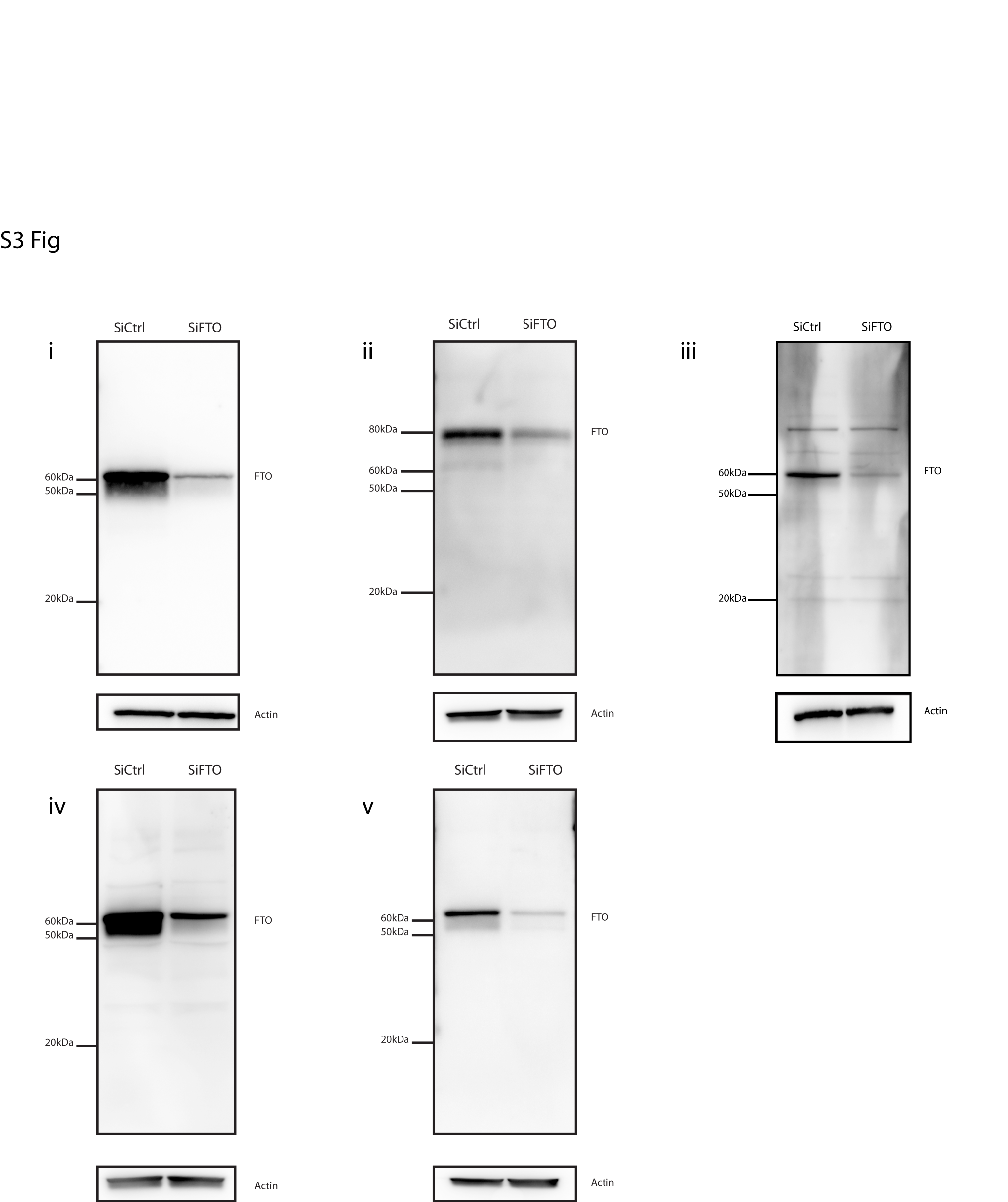

Supplement: S3 Fig — U2OS cells were treated with siControl or siFTO and stained with the antibodies specified in Table 1. (TIF) [file pone.0168182.s003.tif]

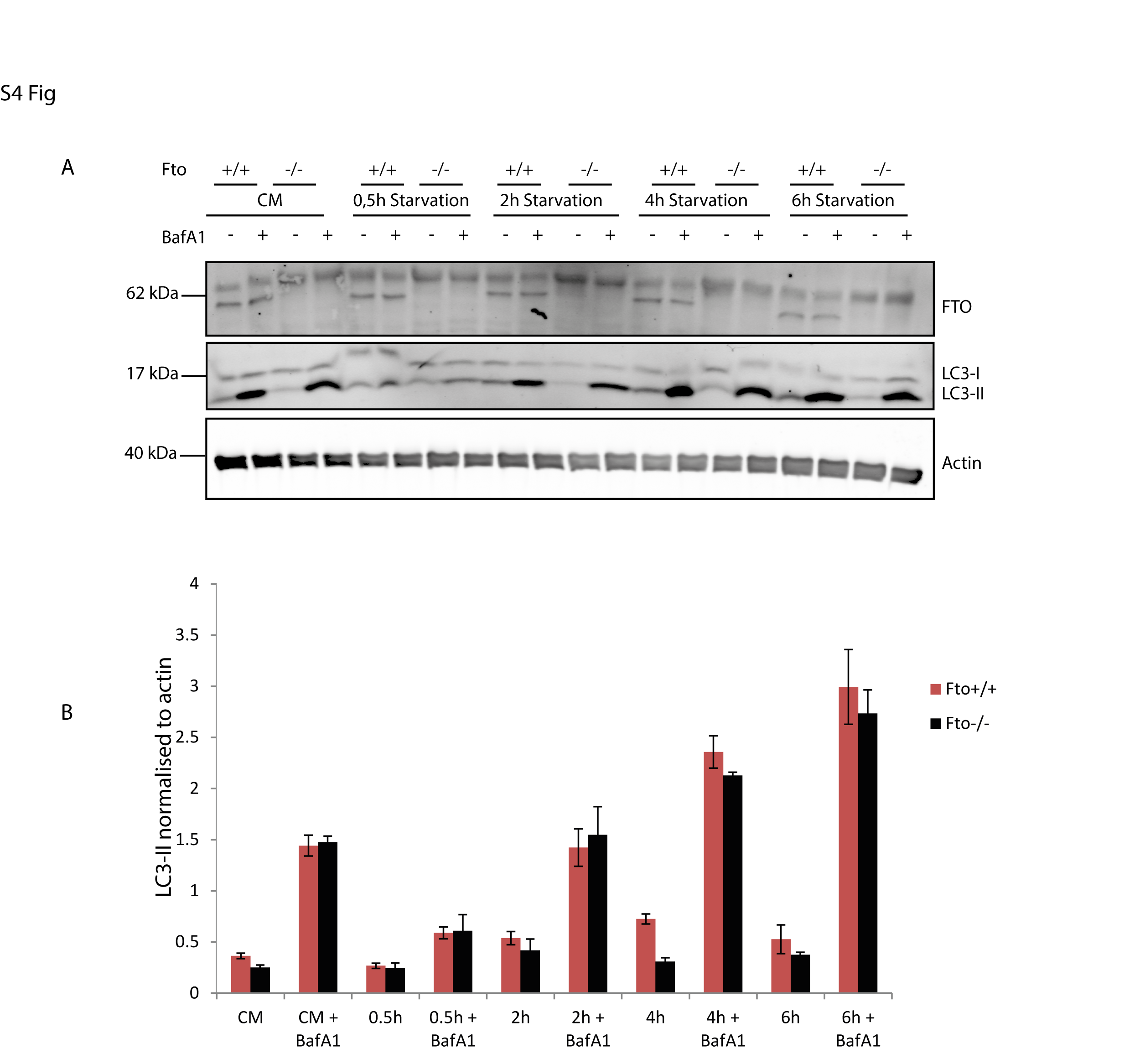

Supplement: S4 Fig — (A) Western blot analysis of MEFs treated with CM or EBSS supplemented with dialysed serum for 0.5h, 2h, 4h, or 6h with or with BafA1. BafA1 was added for 6 hours to the CM treated cells. (B) Quantification of LC3-II levels in MEFs treated with CM or EBSS supplemented with dialysed serum for 0,5h, 2h, 4h, or 6h with or with BafA1. Data are from 3 experiments and presented as mean ± SEM. (TIF) [file pone.0168182.s004.tif]

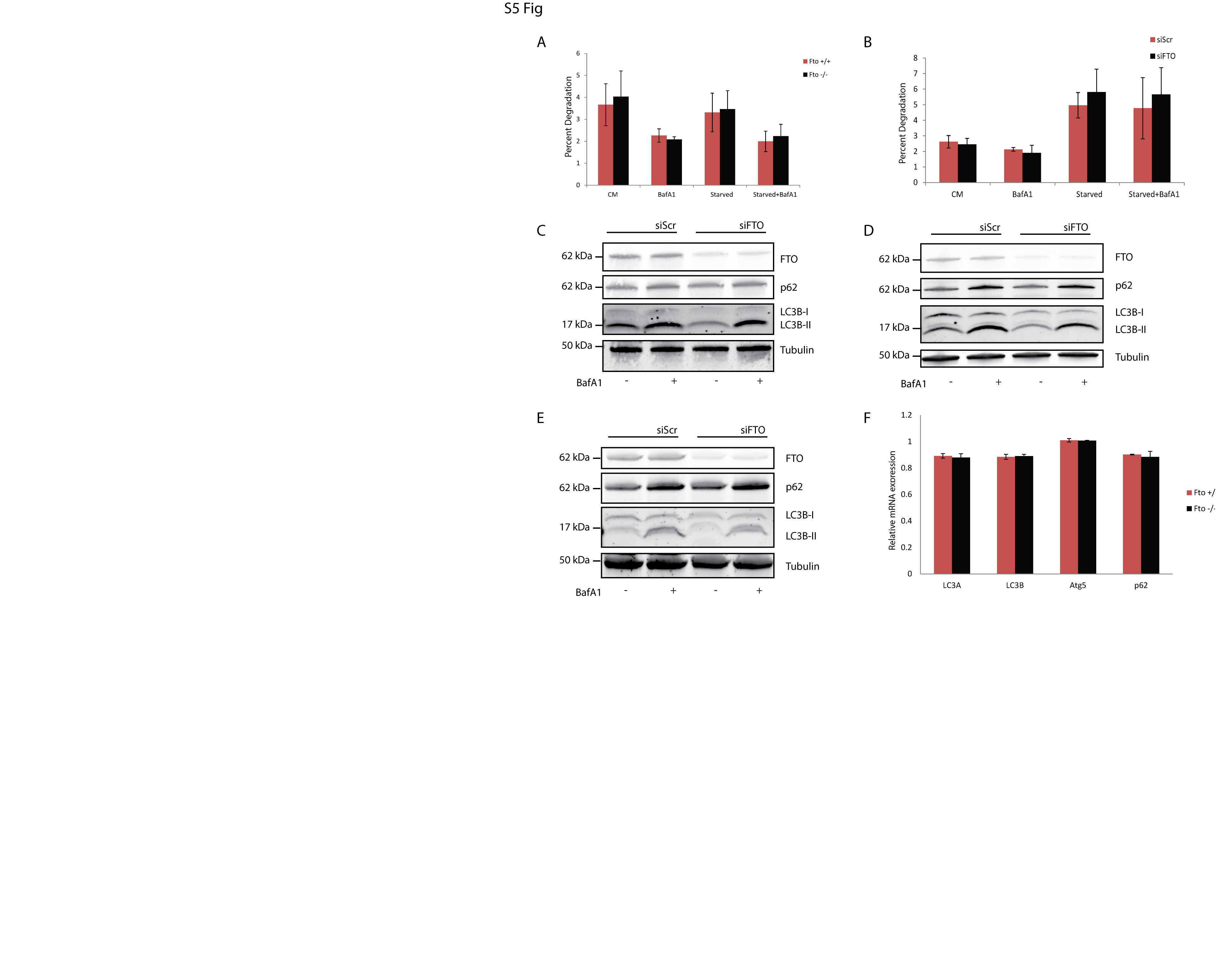

Supplement: S5 Fig — (A) Degradation of long-lived proteins in Fto+/+ or Fto-/- MEFs treated either with complete media (CM), complete media (CM) and Bafilomycin A1 (BafA1), EBSS starvation media (Starved) or EBSS starvation media and Bafilomycin A1 (Starved + BafA1) for 4 hours. The data are from 2 experiments and presented as mean ± SD. (B) Degradation of long-lived proteins in U2OS cells treated as in A. The data are from 2 experiments and presented as mean ± SD. (C) Western blot analysis of protein lysates from control and FTO depleted HeLa cells treated either with complete media in the absence or presence of BafA1 for 4 hours. (D) Western blot analysis of protein lysates from control and FTO depleted U2OS cells treated as in C. (E) Western blot analysis of protein lysates from control and FTO depleted HEK293 cells treated as in C. (F) The graph is showing the relative expression of the denoted targets measured by real-time PCR and normalised to TATA box binding protein (Tbp) in MEFs. Data presented as mean ± SD. (TIF) [file pone.0168182.s005.tif]
